# Structural basis for germline gene usage of a potent class of antibodies targeting the CD4 binding site of HIV-1 gp120

**DOI:** 10.1186/1742-4690-9-S2-O33

**Published:** 2012-09-13

**Authors:** AP West, R Diskin, MC Nussenzweig, PJ Bjorkman

**Affiliations:** 1 California Institute of Technology, Pasadena, CA, USA; 2 The Rockefeller University/Howard Hughes Medical Institute, New York, NY, USA; 3 California Institute of Technology/Howard Hughes Medical Institute, Pasadena, CA, USA

## Background

A large number of anti-HIV-1 antibodies targeting the CD4 binding site (CD4bs) on gp120 have recently been reported. These antibodies, typified by VRC01, are remarkable for both their breadth and potency. Crystal structures have revealed a common mode of binding for several of these antibodies; however, the precise relationship among CD4bs antibodies remains to be defined.

## Methods

We analyze existing structural and sequence data, propose a set of signature features for potent VRC01-like (PVL) antibodies, and test the importance of these features by mutagenesis.

## Results

A group of highly potent CD4bs antibodies, previously isolated from 5 different individuals, all derive from the human VH1-2 gene segment and share a set of characteristic residues, including W50, N58, R71, and W100B. Mutagenesis studies on a half-germline version of a VRC01-like antibody confirm that these signature residues are critical for gp120 binding. Neutralization assays with viruses mutated at sites that contact these critical antibody residues also confirm the significance of these interactions.

## Conclusion

The signature features explain why PVL antibodies derive from a single germ-line human VH gene segment and why certain gp120 sequences are associated with antibody resistance. This analysis also suggests that immunization experiments to elicit VRC01-like antibodies may be problematic in mice or rabbits since they lack germ-line VH genes with all the critical residues. Our results bear on vaccine development and structure-based design to improve the potency and breadth of anti-CD4bs antibodies.

